# Bionomics of the Non-Native Elm Defoliator *Aproceros leucopoda* (Hymenoptera, Argidae) in North-Eastern Italy

**DOI:** 10.3390/insects17040390

**Published:** 2026-04-03

**Authors:** Elena Cargnus, Pietro Zandigiacomo, Francesco Pavan

**Affiliations:** Department of Agricultural, Food, Environmental and Animal Sciences (DI4A), University of Udine, Via delle Scienze 206, 31000 Udine, Italy; pietro.zandigiacomo@uniud.it (P.Z.); francesco.pavan@uniud.it (F.P.)

**Keywords:** invasive pest species, zigzag elm sawfly, *Ulmus*, plant defoliation, number of generations, natural enemies, population dynamics, degree days, photoperiod

## Abstract

*Aproceros leucopoda* (Hymenoptera: Argidae) is an East-Asian pest of *Ulmus* spp. that has spread across Europe since the 2000s and, more recently, to North America, causing repeated defoliation of host plants. Laboratory studies have suggested four or five generations per year in Hungary; however, in the field, their number ranges from one to six. In 2012 and 2013, the bionomics of this invasive pest were studied in north-eastern Italy through weekly samplings from April to October, with data related to accumulated degree days (DDs). Although adult captures exhibited five peaks in both years, only in 2012 were eggs of the fifth and last generation of the season. Their apparent absence in 2013 might be due to elm water stress or temperatures much higher than optimal (30 vs. 19.5 °C). From 2013 to today, a progressive decline in *A. leucopoda* populations has been recorded. The possible reasons for the gradual decline in the sawfly population recorded in the subsequent years are discussed. Several natural enemies were observed. This study can contribute to a better understanding of *A. leucopoda* population dynamics in newly colonised areas, including the risk to wood production in mixed deciduous plantations if defoliation occurs over many consecutive years.

## Simple Summary

The East Asian zigzag elm sawfly is a pest that feeds on elm trees and can cause severe defoliation in urban areas and mixed forest plantations. Over the past 20 years, it has spread across Europe and has recently been detected in North America, raising concerns about the health of elms. In north-eastern Italy, this invasive species was first detected in 2009. Its life history in newly invaded areas is not fully known. This study investigated the phenology and the number of generations per year the sawfly develops over the vegetative season in north-eastern Italy. Weekly field sampling over two years (2012–2013) showed five peaks of adult captures, suggesting five generations. However, in 2013, no eggs were found for the final generation, probably due to very high summer temperatures and drought that stressed the elm trees. From 2013 to today, a progressive decline in *A. leucopoda* populations has been recorded. Several natural enemies were observed during the study. These findings can help predict future outbreaks and support better management of elm trees in cities and mixed forest plantations.

## 1. Introduction

The invasive alien zigzag elm sawfly, *Aproceros leucopoda* Takeuchi (Hymenoptera, Argidae), is a pest that feeds on elm leaves (Ulmaceae) native to East Asia [1]. After its first detection in Central and Eastern Europe in the early 2000s [1,2,3], the species has spread to most European countries, including Italy [4,5,6,7], Western Europe, the Balkans, and the European part of Russia [8,9,10,11,12,13,14,15,16,17,18,19,20,21,22,23,24,25]. The last and southernmost European country where the species has been recorded is Greece [26]. Since the 2020s, it has also been detected in North America, including Canada and several eastern U.S. states [27,28,29,30,31,32].

The sawfly mostly attacks various species and cultivars of elms (*Ulmus* spp.), including those grown in nurseries for planting as ornamental trees, along roads, in gardens, parks, and in lowland mixed deciduous plantations for wood production [1,3,12,27,29,32,33,34,35]. In Europe, damage has been reported for *U. glabra* Hubs., *U*. *davidiana* Planch., *U. laciniata* (Trautv.) Mayr, *U. laevis* Pall., *U. minor* Mill., *U. pumila* L., and *U. pumila* var. *arborea* Litv. [1,4,34].

In Italy, specifically in the north-eastern Friuli Venezia Giulia region, the most severe damage was observed on the Siberian elm *U. pumila* and the field elm *U. minor* [4]. According to the study by Vétek et al. [36], it cannot complete development on the Ulmaceae *Hemiptelea davidii* (Hance) Planch and *Zelkova serrata* (Thunb.) Makino, although the latter has recently been reported as an alternate host during the early vegetative season [32]. The species reproduces by thelytokous parthenogenesis, overwinters in the eonymphal stage, and develops multiple generations per year [1]. Rapid population expansion, associated with severe elm defoliation, can occur in both ornamental plantings and mixed deciduous plantations [1,4,17,35,37,38]. Repeated defoliation by the sawfly not only reduces the aesthetic value of trees and wood production [32], but it may also increase the plant’s susceptibility to Dutch elm disease and elm yellows, which are associated with bark beetles (*Scolytus* spp.) and leafhoppers as pathogen vectors, respectively [39,40,41,42].

On elms, females lay eggs along leaf margins. Newly hatched larvae feed on leaves in a characteristic zigzag pattern, while later instars consume leaves nearly completely. The six-instar larvae spin cocoons and pupate [1,35]. Not overwintering cocoons are light-silken, loosely spun, and mainly attached to the lower surface of leaves, twigs, and other woody parts; diapausing cocoons are brown, double-walled, and are found in leaf litter or the topsoil layer [1,17,35,37,43]. Adults can actively disperse 45–90 km per year [1,3].

Insecticide application was successful against first-generation larvae in Hungary; however, because adults can quickly reinvade a treated area, local spraying appears unlikely to be an effective method for controlling the pest [1]. In a field study conducted in Romania, *Beauveria bassiana* (Bals.) Vuill. applied in mixed-forest plantations against *A. leucopoda* resulted in a decrease in defoliator densities [44]. Occasional reports exist of both generalist predators and parasitoids [1,11,12,35,45,46,47,48,49,50,51].

In the Palaearctic region, the number of *A. leucopoda* generations was at least two in Poland [1], three in the Czech Republic [17], up to four in Italy, Hungary, Japan, and China [1,4], two to four in Russia [9,13,35,47], possibly up to six in the Netherlands [16]. In North America, one or two generations in Virginia [29], and up to five generations in North Carolina [32] were reported. In the laboratory, it was estimated that *A. leucopoda* might develop through up to four or five generations per year in Hungarian climatic conditions [37].

This two-year study, conducted in north-eastern Italy, aims to contribute to the understanding of the basic biology of this invasive species by providing the first data on its phenology, which is essential for determining the number of annual generations developed by the sawfly in the two years. Phenological patterns are assessed in relation to mean annual temperature, photoperiod, and degree day accumulation. Because elm damage can occur in nurseries and mixed deciduous plantations, it is important to focus on the life history of this economically important pest species.

## 2. Materials and Methods

### 2.1. Study Sites

A two-year study (2012–2013) was conducted at two locations of lowland agroecosystem in the Udine district (UD), in north-eastern Italy. Site 1 (loc. Reana del Rojale, 46°8′21.51″ N, 13°13′17.57″ E, 162 m a.s.l.) was a 100 × 80 m plantation of mixed *U. pumila* and *Robinia pseudoacacia* L. trees. Site 2 (loc. Martignacco, 46°5′6.00″ N, 13°10′8.56″ E, 122 m a.s.l.) included a 170 m ornamental row of *U. pumila* trees close to 150 × 80 m mixed *U. pumila* and *R. pseudoacacia* grove. Both sites, 7 km apart, shared similar climatic conditions and experienced severe damage from *A. leucopoda* the year preceding the study.

### 2.2. Preliminary Site Survey in Early Spring

In each study year, before starting regular sampling, *U. pumila* trees at sites 1 and 2 were inspected at least once to select those most suitable for this study and to assess their vegetative development. During inspections, visual observations were conducted to detect the start of emergence of *A. leucopoda* adults of the overwintering generation.

### 2.3. Monitoring with Traps, Sampling on Leaves, and Phenology

For monitoring *A. leucopoda* flight, a fluorescent yellow (PALz) “cloak” trap with a sticky surface measuring 23 × 36 cm (Csalomon^®^, Plant Protection Institute, Centre for Agricultural Research, Hungarian Academy of Sciences, HUN-REN, Budapest, Hungary, www.csalomontraps.com, accessed on 20 April 2025) was utilised, as suggested in Vétek et al. [52]. At both years and two study sites, two traps 10 m apart were installed on 12 April. The traps were hung with wires from the lower branches of trees, at a height of 1.5–2.0 m, and inspected weekly until the end of October, to ensure coverage of the entire adult flight period. At each trap check, captured individuals were counted and removed.

Concurrent with trap installation and inspection dates, eggs, larvae, and pupae of *A. leucopoda* were sampled on elm leaves in site 1 in 2012 and site 2 in 2013. Although trapping was carried out at both sites in both years, immature-stage sampling in 2013 was not feasible at site 1 due to a reduced elm tree availability. The two study sites may be considered interchangeable, as they are located within the same geographic area (10 km apart) and affected by similar levels of foliage damage. In confirmation of this, during 2012, when systematic sampling was conducted at site 1, additional weekly sampling was also performed at site 2 (12 April–24 May and 9 August–30 September), with no differences found between the two sites. Trees and shoots were chosen randomly, and only fully expanded leaves were examined. At each sampling, 500 leaves, from branches at a height of 1.5–2 m (i.e., approximately human height), were sampled on site from early April to late September. The number of eggs, larvae, and pupae present on each leaf was recorded. Larvae were also subdivided by body size into early (1st–2nd instar), middle (3rd–4th instars), and late (5th–6th instars), and pupae by colour into green (newly formed pupae) and black (older pupae, with morphological characteristics of adults). Larvae and pupae belonging to different types were identified in the field according to G. Vétek [53]. The early larvae are 1–2 mm in size and create zigzag feeding patterns, starting from the leaf margin (where the females lay eggs) and continuing between two leaf veins toward the midrib. The middle larvae (4–5 mm in size) eat the entire leaf between the two veins, making the zigzag pattern less evident. The late larvae (7–8 mm in size) consume the entire leaf, making it skeletonized. During sampling, the presence of double-walled cocoons on leaves was recorded, as well as any larvae dropped to the ground. Field-collected data on eggs, larvae, pupae, and adults of *A. leucopoda* were used to describe their phenology and establish the number of generations per year. The criteria adopted to investigate the voltinism of this species are illustrated in Table S1. To establish the phenology of the different generations, literature data on the pre-oviposition period and the developmental durations of eggs and larvae were also used [37]. To compare field data with available laboratory data, the cumulated degree days (DDs) corresponding to specific thermal constants (K), such as first adult emergence or generation development time, were calculated from 1st January, assuming a lower thermal threshold of 7 °C, as estimated by Papp et al. [37]. When two stages of the same generation (e.g., eggs and first-instar larvae) were first observed on the same sampling day, the onset of the earlier stage was estimated to occur at the intermediate date between the current and previous sampling.

Weather data were obtained from a weather station located in Udine (named Udine S.O., 46°2′6.82″ N, 13°13′36.07″ E, 91 m a.s.l., ARPA-OSMER http://www.osmer.fvg.it/ accessed on 7 May 2025) situated within 10 km south of the two studied sites (Table S2 and Figure S1).

### 2.4. Notes on Natural Enemies

At the leaf samplings, arthropod natural enemies of *A. leucopoda* were recorded and collected at both study sites. Immatures were reared in the laboratory, and adults were identified to species or genus level using available taxonomic keys [54,55,56,57,58]. Furthermore, specimens identified by specialists and present in the entomological collections of Agricultural, Food, Environmental, and Animal Sciences (DI4A), University of Udine, were examined for comparison.

## 3. Results

### 3.1. Preliminary Survey of Study Sites

On 2 April 2012, early leaf emergence was observed on elm trees at both study sites. Rainy weather prevented site inspections for one week (Figure S1). By 12 April, the date of trap installation, the leaves had developed further. In 2013, at both study sites, a few elm leaves had developed during the first week of April.

### 3.2. Adult Monitoring

**Figure 1:**
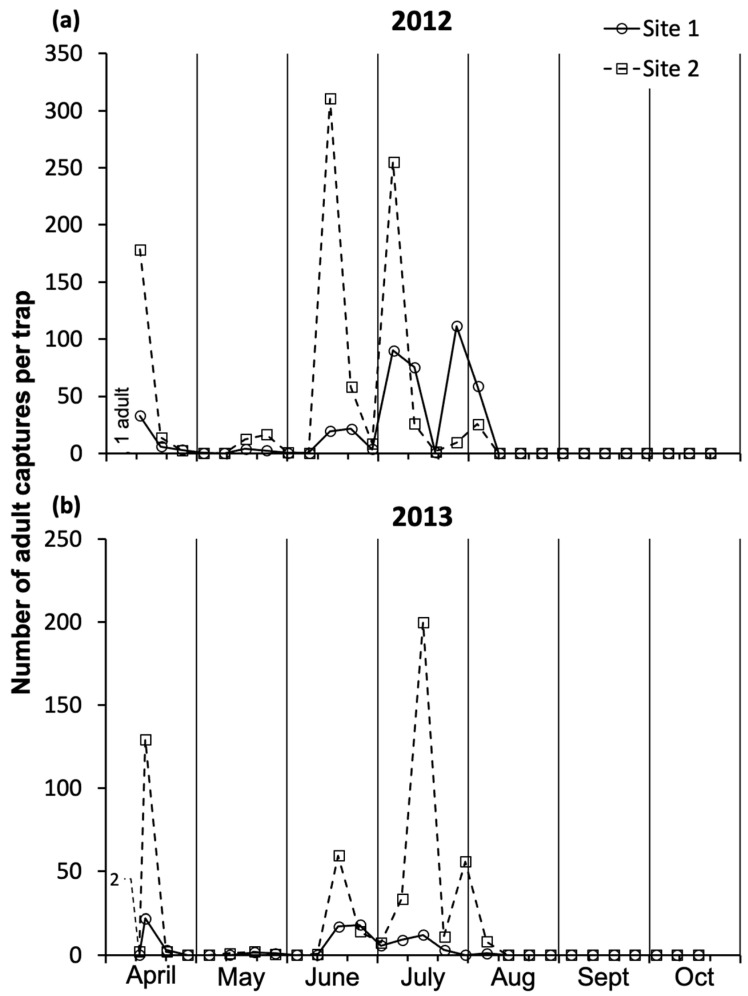
Weekly captures of *Aproceros leucopoda* adult females at study sites 1 and 2 using sticky traps, from early April to late October in (**a**) 2012 and (**b**) 2013. In (**a**), the vertical text refers to visual observation of an adult. The sum and average of adults are presented in Table S3.

In both study years, all recorded *A. leucopoda* adults were female. In 2012, adults were visually recorded or captured on traps from early April to early August (Figure 1a; Table S3). During the first visual inspection of the study sites (2 April), a single female was observed. The first adult peak occurred in mid-April, followed by four further distinct peaks in late May, mid–late June, early mid-July, and late July–early August. At the late-May peak, the captures were the lowest at both sites. From mid-August to late October, no adults were captured. Excluding the last peak, the captures were higher at site 2 than at site 1.

In 2013, *A. leucopoda* adults were visually recorded or captured on traps from mid-April to mid-August (Figure 1b; Table S3). During the first visual inspection of the study sites (2 April), no females were observed. The first two females of the season were recorded on 12 April at site 2. At both sites, after the first adult peak in mid–late April, a few captures were recorded in late May and two other peaks, respectively in mid-June and mid-July. In late July–early August, the last peak was recorded only at site 2, while only one capture occurred at site 1. From mid-August to late October, no adults were captured.

### 3.3. Immature Stage Sampling on Leaves

**Figure 2:**
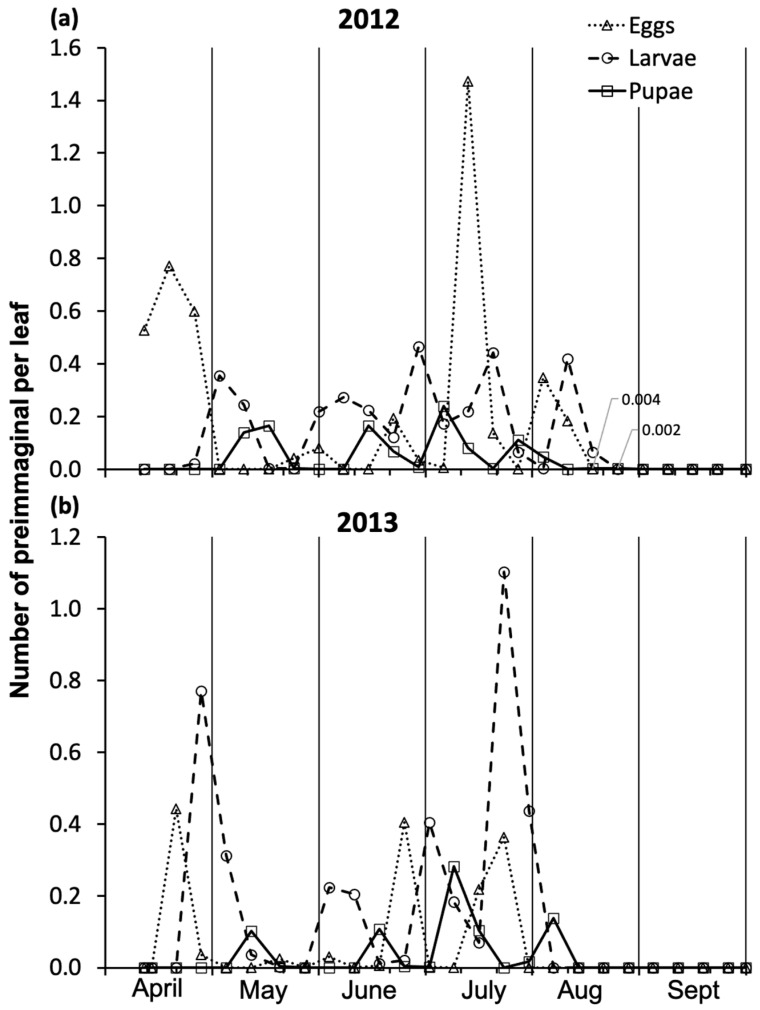
*Aproceros leucopoda* eggs, larvae, and pupae recorded weekly on 500 leaves of *Ulmus pumila*, from early April to late September in (**a**) 2012 and (**b**) 2013, at study sites 1 and 2, respectively. The values 0.004 and 0.002 shown in the upper graph correspond to the pupal stage. For each immature stage, the sum, average, and standard deviation are presented in Table S4.

In 2012, at the site under leaf sampling (site 1), *A. leucopoda* eggs were first recorded on the initial spring sampling date (on 12 April), indicating ongoing oviposition by overwintering-generation females (Figure 2a; Table S4). Five egg peaks were recorded in mid-April, late May, mid-June, mid-July, and early August, with the fourth being the highest. No more eggs were recorded from mid-August until the last sampling date. The first newly hatched larvae, associated with an initial zig-zag track of about 1–2 mm on the leaves, were observed on 26 April. Then, larvae were present until mid-August, with five peaks of similar magnitude in early May, late June, mid-July, and early August, following those of the eggs. No larvae were observed from late August until late September. Pupae were first observed during early May sampling. Their number increased, with evident peaks in mid-May, mid-June, early July, and late July, following the respective larval peaks. Pupae were present at a similar population level across these periods. After the late-July peak, rare pupae were observed in August (i.e., two with newly formed pupae on 16 August and one older pupa on 23 August), and none were observed until late September. The data recorded at site 2, over the two intervals 12 April–24 May and 9 August–30 October, were consistent with those collected at site 1.

In 2013, at the site under leaf sampling (site 2), *A. leucopoda* eggs were first recorded during the second weekly sampling (on 21 April), when the first and highest peak of the season occurred (Figure 2b; Table S4). Between early May and early June, eggs were recorded on only two isolated sampling dates. Two egg peaks were recorded in late June and late July. No eggs were observed from late July to the end of September. Larvae were present from late April to late July, with four peaks, i.e., in late April, early June, late June, and mid–late July, all after egg occurrence or peaks. The first and last larval peaks were more than twice as high as the two intermediate peaks. On 29 July 2013, approximately 30 last instar larvae were seen falling to the ground onto a 1 m^2^ surface within 10 min of observation. No larvae were recorded from early August to late September. Pupae were recorded from mid-May to early August, with four peaks in mid-May, mid-June, early July, and early August, each following a larval peak. Pupae density level was highest in early July and similar across the other periods. The level of pupal peaks was much lower than that of the corresponding larval peaks, especially for the first and fourth peaks.

In both years, some double-walled cocoons on leaves were observed at the two study sites as early as July.

**Figure 3:**
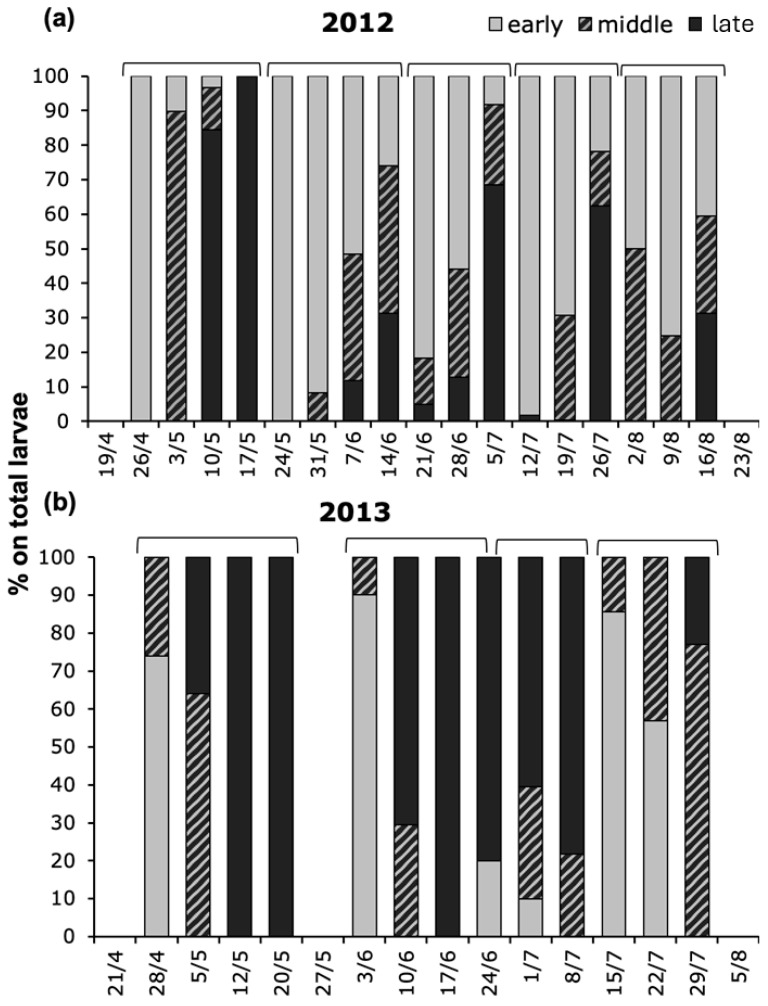
Proportion of *Aproceros leucopoda* larvae, recorded weekly on 500 leaves of *Ulmus pumila* in (**a**) 2012 and (**b**) 2013, at study sites 1 and 2, respectively, subdivided into early (1st–2nd instars), middle (3rd–4th instars), and late (5th–6th instars). The sum of larvae is presented in Table S5.

In 2012, five sampling dates with the first appearance of early larval instars were followed by samplings with a progressive increase in older instars (Figure 3a; Table S5). In 2013, four periods were recorded in which the prevalence of early-instar larvae was followed by a progressive increase or the exclusive presence of older-instar larvae (Figure 3b; Table S5).

**Figure 4:**
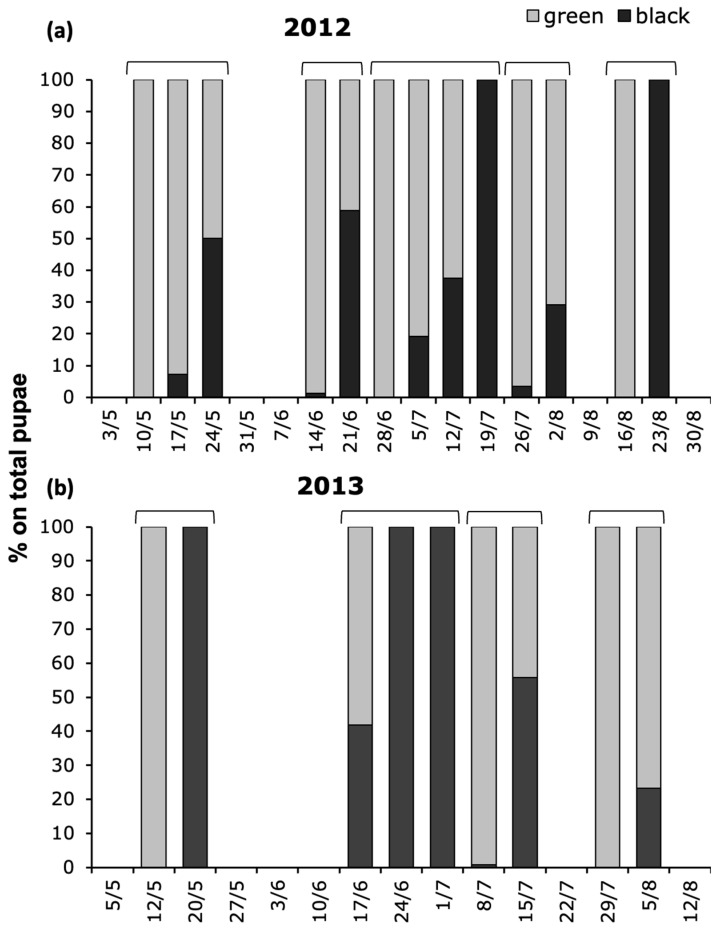
Proportion of *Aproceros leucopoda* pupae, subdivided into newly formed pupae (which are light-green individuals) and older pupae (which are black individuals, resembling adults), inside the summer cocoons, recorded on 500 leaves of *Ulmus pumila* in (**a**) 2012 and (**b**) 2013, at study sites 1 and 2, respectively. The sum of pupae is presented in Table S5.

In 2012, five sampling dates with a prevalent or exclusive presence of green pupae (i.e., newly formed) were followed by one or two sampling dates with a higher proportion of black pupae (i.e., older, that resemble adults) (Figure 4a; Table S5). In 2013, four periods were evident in which newly formed pupae were replaced by a higher proportion of older pupae (Figure 4b; Table S5).

### 3.4. Life Cycle and Generations

**Figure 5:**
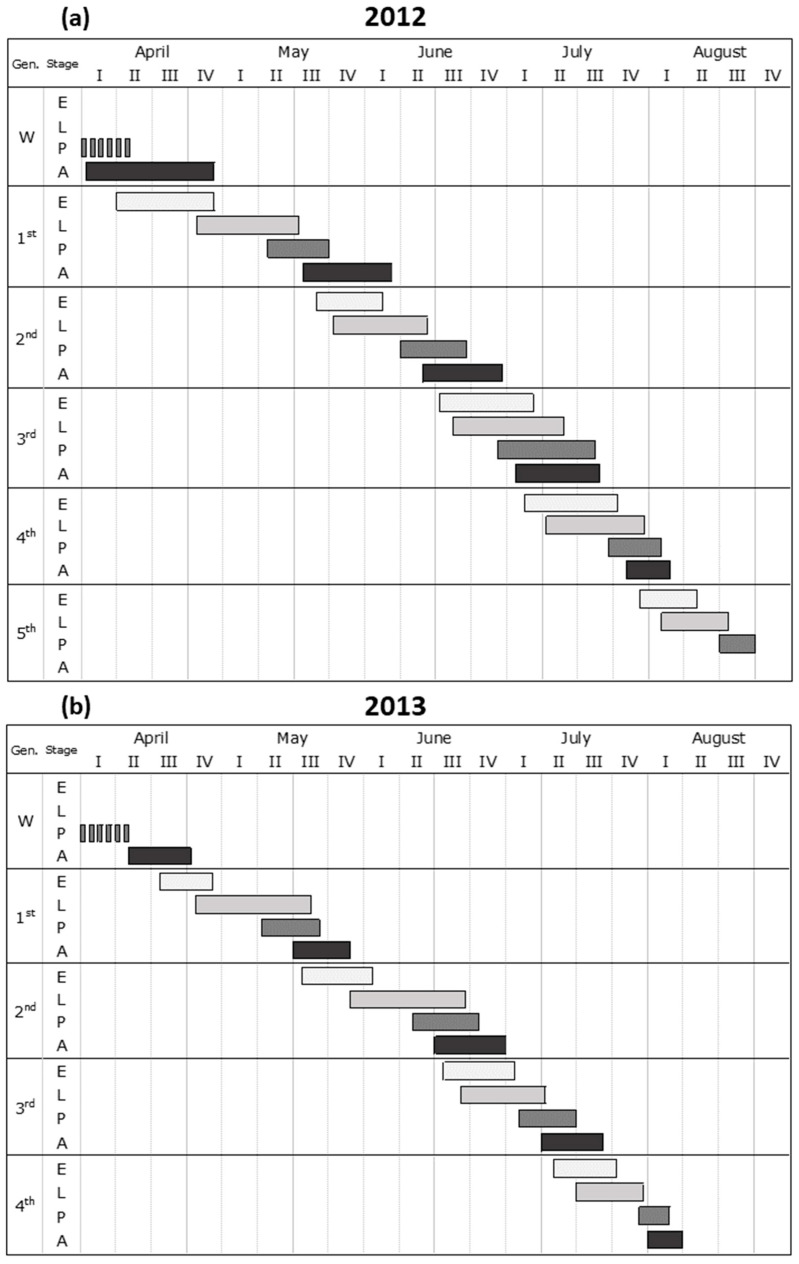
Phenological data of *Aproceros leucopoda* in (**a**) 2012 and (**b**) 2013. Eggs (E), larvae (L), pupae (P), and adults (A) were collected through weekly sampling at study sites 1 and 2. Gen.: generation; W: wintering. Shaded bars indicate the pupal stage of the species, which overwinters in the soil (data not recorded during this study), from which adults emerge in the spring.

Based on field observations, in 2012, *A. leucopoda* developed five generations, while in 2013 the species apparently completed only four generations, as eggs of a fifth generation were not recorded (Figure 5a,b).

The degree days (DDs) associated with each generation are reported in Table 1. In 2012, the emergence of overwintering-generation adults and the subsequent onset of egg laying occurred earlier than in 2013, although this required more DD accumulation. From the hatching of the first-generation eggs to the pupae of the third generation, accumulated DDs remained approximately 100 DDs higher in 2012 than in 2013, despite phenological phases occurring on similar calendar dates in both years. From third-generation adults to fourth-generation adults, the differences in DD accumulation decreased, becoming almost the same in the two years. In both years, the DDs required for the development of a generation increased from the first to the fourth generation, resulting in lower values for all generations than the 426 DDs, calculated in the laboratory [37]. Comparing the two years, the number of DDs required to develop a generation (from eggs to eggs) was slightly higher in 2012 than in 2013 for the first (+6.6 DDs) and second (+16 DDs) generations, while the opposite occurred for the third (−32.7 DDs) generation.

### 3.5. Natural Enemies

On elms at the two study sites, a range of arthropod predators and parasitoids were observed attacking *A. leucopoda* in both study years, mainly during the June–July period.

Among predators, hemipteran nymphs of the true bugs *Arma custos* (Fabricius), *Pinthaeus sanguinipes* (Fabricius), and *Zicrona caerulea* (L.), as well as those of the nabid bug *Himacerus apterus* (Fabricius), were observed consuming *A. leucopoda* pupae within cocoons on elm leaves. *Aproceros leucopoda* larvae were attacked by ants and spiders. Under infested elm trees, numerous carnivorous ants of the species *Formica cunicularia* Latreille (Hymenoptera) were observed carrying sawfly larvae, which had fallen to the ground, between their mandibles. On leaves, the spiders *Enoplognatha ovata* (Clerck) (Theridiidae) and *Xysticus* sp. (Thomisidae) (Araneae) fed on sawfly larvae.

On elm leaves, two cocoons with a single tachinid parasitoid (Diptera) pupa, along with a sawfly eonymph, were observed. From this material, two adult tachinid specimens emerged in the laboratory, but only one was identified as *Blondelia nigripes* (Fallén), since the diagnostic characters were not visible in the other.

Other potential generalist predators, i.e., not feeding on the pest at the time of the observation, included the hemipteran mirids *Heterotoma merioptera* Scopoli, *Deraeocoris ruber* (L.), and *Deraeocoris lutescens* (Schilling), as well as chrysopid *Chrysopa* sp. (Neuroptera) and coccinellid *Harmonia axyridis* (Pallas) (Coleoptera).

## 4. Discussion

### 4.1. Lifecycle of A. leucopoda on U. pumila

The results of this study indicate that, under north-eastern Italian conditions, *A. leucopoda* develops four to five generations per year. In both 2012 and 2013, the adult females’ flight period ranged from early to mid-April through early August, with five distinct peaks, consistent with five generations. However, immature-stage sampling allowed us to ascertain the development of the fifth generation only in 2012. In fact, in 2013, no eggs or larvae were observed after the fifth adult peak, suggesting that the fifth generation may not have developed. If a fifth generation did occur, the fact that it was not observed may indicate that environmental factors led to a population collapse.

Hungarian data support our observations about the first adult peak in mid-April and the occurrence of at least four generations per year [1]. In Japan, the flight period was delayed by about a month compared to the present study, occurring from mid-May to early September; nonetheless, four generations were reported [1]. Similarly, in Ohio (USA), the adult flight occurred from mid–late April to early September, i.e., slightly later than in Italy, with five generations reported [32]. In contrast, in the Czech Republic, individuals recorded in early September belonged to larval and pupal stages of the third generation, suggesting more limited voltinism [17]. In the Azov and Rostov regions, the onset of adult flight was reported approximately one month later than in the current study (i.e., starting in mid-May), resulting in three generations completed by late July [9]. However, for the same regions and for the nearby Donetsk one, other studies reported sawfly phenology similar to that of the present study in 2013, with the development of four generations. [35,47]. In the Netherlands, compared to north-eastern Italy, adult flights started two weeks later (late April) but ended a month later (mid-September), while larvae were recorded until two months later (mid-October), suggesting the potential for up to six generations in the former country [16].

In spring, later adult emergence in the Netherlands compared to northern Italy ([16] vs. the present study) is likely due to lower temperatures in northern Europe (April minimum: 6 °C in Amsterdam vs. 9 °C in Udine; maximum: 14 °C vs. 19 °C, respectively; https://weather-and-climate.com, accessed on 23 May 2025). Conversely, late summer-early autumn temperatures cannot account for the longer occurrence of the sawfly in the feeding activity in the Netherlands, because they drop earlier there (September minimum: 13 °C in Amsterdam vs. 15 °C in Udine; maximum: 19 °C vs. 25 °C, respectively; https://weather-and-climate.com; accessed on 23 May 2025). The development of the sawfly until autumn only in the Netherlands could have two explanations associated with two features of the climate of northern Italy: (i) the shorter photoperiod may induce an earlier diapause; (ii) summer temperatures (Table S2; Figure S1), much higher than the optimum (19.5 °C [37]), may cause population collapse.

The flight of adults from the overwintering generation started earlier in 2012 (2 April) than in 2013 (12 April), but in 2012, more DDs were needed for emergence. This suggests that the number of accumulated DDs from January 1st is not a reliable predictor of the timing of adult emergence. DDs proved useful for estimating the number of generations starting from the beginning of oviposition by the first generation. However, the DDs required per generation increased from the first to fourth generation and were lower for the first three generations and similar for the fourth compared to DDs calculated under laboratory conditions [37]. The higher DDs in the third generation, especially in 2013, may be explained by crossing the upper thermal threshold during the hotter summer temperatures (see Table S2; Figure S1). Furthermore, in 2013, excessively high temperatures may have impaired female fecundity (in July: mean of 24.4 °C in 2012 vs. 25.6 °C in 2013; see Table S2; Figure S1), as suggested by a laboratory study by Papp et al. [37]. At the beginning of the emergence of the fourth-generation females (i.e., 26 July 2012 and 1 August 2013), the shorter photoperiod in 2013 (14:47 h vs. 15:03 h in 2012) may also have contributed to the absence of observed eggs. Additionally, increased plant stress due to higher temperatures and lower rainfall in 2013 (see Table S2; Figure S1) may have reduced leaf quality and the production of new foliage, limiting infestation by the sawfly as females prefer soft and younger leaves to thicker and older ones for oviposition ([4,13,47], this study). These environmental conditions may also explain the dropping of mature larvae to the ground, and likely spun the cocoon in the soil to overwinter, since it is known that the three factors considered (food quality, high temperatures, and short photoperiod) can induce diapause in insects [59,60]. The dropping of the larvae explains why the number of pupae of the last generation observed on the leaves was much lower than the number of larvae of the same generation.

The presence of double-walled cocoons as early as July suggests that overwintering cocoons are produced throughout the season, in agreement with other studies [1,27,47,48].

### 4.2. Natural Enemies

The stink bugs *P. sanguinipes* and *Z. caerulea*, the spiders *E. ovata* and *Xysticus* sp., and the ant *F. cunicularia* were recorded feeding on *A. leucopoda* for the first time. Additional records of predators (*A. custos* and *H. apterus*) observed feeding on the sawfly immatures, as well as the emergence of the parasitoid (*B. nigripes*) from a sawfly cocoon, confirm previous reports [1,12,46,47,48]. In agreement with other studies, birds were also observed feeding on sawfly larvae, suggesting that both invertebrate and vertebrate natural enemies may contribute to the control of this species (e.g., [35,47]).

### 4.3. Population Decline

As observed for other hymenopteran defoliators [61,62], a decline in populations has also been recorded for *A. leucopoda* in our region (Friuli Venezia Giulia). In fact, following the population collapse in the summer of 2013, the decline was observed at the regional level in forest plantations [63], including several sites previously reported as severely infested [4]. Similarly, in Traismauer (Austria), the population of *A. leucopoda*, after reaching pest status in 2009–2010, declined until it became rare in 2013 [11].

Although during the study years, natural enemies appeared unable to control *A. leucopoda* populations at low densities, the decline of their populations in the subsequent years [63] may reflect a gradual adaptation of some native natural enemies to regulate sawfly populations at levels low enough to avoid severe elm damage. However, the role of entomopathogens in this decline is probably more important [47]. In any case, the phenomenon recorded for *A. leucopoda* is common among many defoliators, which have population cycles characterised by a few years of high abundance followed by many years in which they are nearly absent [61,62,64]. Therefore, we cannot exclude the possibility that *A. leucopoda* populations may return to high levels in the coming years.

### 4.4. Economic Importance

In the first years after its introduction, *A. leucopoda* can cause severe defoliation of elms in nurseries, urban plantings, and mixed deciduous plantations in many of the areas where the species has been newly detected ([1,4,12,17,29,32,34,35,36], this study). At present, tree mortality has not been reported, but repeated defoliations year after year compromise the tree’s health and aesthetic value. Fortunately, a subsequent reduction in the sawfly population density seems to have averted this outcome, at least within the study area. Trees attacked by *A. leucopoda* often show a secondary bud burst, but feeding on new leaves can cause twig and branch dieback and reduced growth [1,4]. However, if population decline does not occur, appropriate control strategies will need to be developed. In this context, studies on the potential role of natural enemies in the control of *A. leucopoda* can also be useful.

## 5. Conclusions

This study provides new insights into the phenology of *A. leucopoda*. The data on the last occurrence of immature stages in mid-summer, and the population decline observed over the years, may be useful for understanding the phenomena that will occur in newly introduced areas. This information will be useful because the broad adaptability to low temperatures suggests that the geographic distribution of the sawfly may continue to expand.

## Figures and Tables

**Table 1:** Number of degree days (DDs) (thermal accumulation) and calendar dates (dd/mm) associated with the first occurrence of *Aproceros leucopoda* eggs (E), larvae (L), pupae (P), and adults (A) during the vegetative season in 2012 and 2013, respectively. In the last two columns, DDs for developing one generation, from egg to egg, are reported. Based on the laboratory study of Papp et al. [37], the lower thermal threshold for calculating DDs was 7 °C, and the reference thermal constant for completing a generation was 426 DDs. Gen.: generation; *: first adults observed in the field.

Gen.	Period	DDs and Date (dd/mm) of First Occurrence of Each Stage								DDs of One Gen.	
		E		L		P		A		E-E	
		2012	2013	2012	2013	2012	2013	2012	2013	2012	2013
*	1 January to early April							160.7 2/4	69.3 12/4		
1	early April to late May	189.7 8/4	113.8 17/4	261.9 26/4	199.4 26/4	414.9 10/5	346.3 9/5	474.2 17/5	391.3 14/5	311.0 42	304.4 30
2	mid-May to early July	500.7 20/5	418.2 17/5	543.8 24/5	496.4 28/5	738.7 8/6	638.4 11/6	795.8 13/6	701.5 15/6	336.6 27	320.0 31
3	mid-June to late July	837.3 16/6	738.2 17/6	893.5 19/6	817.2 21/6	1075.5 29/6	996.5 4/7	1157.4 3/7	1071.7 8/7	358.8 19	391.5 24
4	early July to early August	1196.1 5/7	1129.7 11/7	1267.2 9/7	1200.3 15/7	1491.6 23/7	1473.9 29/7	1541.6 26/7	1528.5 1/8	419.4 25	
5	late July to late August	1615.5 30/7	-	1710.2 4/8	-	1923.1 16/8	-	-	-		

## Data Availability

The original contributions presented in this study are included in the article/Supplementary Materials. Further inquiries can be directed to the corresponding author.

## Supplementary Materials

The following supporting information can be downloaded at: https://www.mdpi.com/article/10.3390/insects17040390/s1, Table S1: Criteria used to define the phenology and number of generations of *Aproceros leucopoda* based on field observations of eggs, larvae, pupae, and adults, supported by published developmental data; Table S2: Mean monthly temperature and rainfall data from April to September (2012–2013) obtained from a weather station located near the study sites 1 and 2 (weather station Udine S.O., https://www.osmer.fvg.it/clima.php?ln=, accessed on 7 May 2025); dd: number of days with daily rainfall greater than or equal to 5 mm/1 mm; Figure S1: Daily temperature and rainfall from April to October in (a) 2012 and (b) 2013, obtained from a weather station located near the study sites 1 and 2 (weather station Udine S.O., https://www.osmer.fvg.it/clima.php?ln=, accessed on 7 May 2025); Table S3: Number of *Aproceros leucopoda* adults captured on two sticky traps per sampling site and date in 2012 and 2013; Table S4: Number of *Aproceros leucopoda* eggs, larvae, and pupae recorded, as total and average with standard deviation (SD) on 500 elm leaves per sampling site and date in 2012 and 2013; Table S5: Proportion of *Aproceros leucopoda* larvae, subdivided into early, middle, and old instars, and pupae, subdivided into early and old pupae, corresponding to newly formed pupae of green colour and older pupae of black colour, respectively, recorded per sampling site and date in 2012 and 2013.
