# Supplementary material for: Bionomics of the Non-Native Elm Defoliator Aproceros leucopoda (Hymenoptera, Argidae) in North-Eastern Italy
Source: Insects. 2026 Apr 3;17(4):390. doi: 10.3390/insects17040390 (PMC13116034; doi:10.3390/insects17040390)
Supplement: Supplementary file 1 [file insects-17-00390-s001.zip › insects-4200153-supplementary.pdf]

insects

MDPI

Supplementary Materials: The following supporting information can be downloaded at: https://www.mdpi.com/article/doi/s1, Table S1: Criteria used to define the phenology and number of generations of *Aproceros leucopoda* based on field observations of eggs, larvae, pupae, and adults, supported by published developmental data; Table S2: Mean monthly temperature and rainfall data from April to September (2012–2013) obtained from a weather station located near the study sites 1 and 2 (weather station Udine S.O., https://www.osmer.fvg.it/clima.php?ln=, accessed on 7 May 2025); dd: number of days with daily rainfall greater than or equal to 5 mm / 1 mm; Figure S1: Daily temperature and rainfall from April to October in **a)** 2012 and **b)** 2013, obtained from a weather station located near the study sites 1 and 2 (weather station Udine S.O., https://www.osmer.fvg.it/clima.php?ln=, accessed on 7 May 2025); Table S3: Number of *Aproceros leucopoda* adults captured on two sticky traps per sampling site and date in 2012 and 2013; Table S4: Number of *Aproceros leucopoda* eggs, larvae, and pupae recorded, as total and average with standard deviation (SD) on 500 leaves per sampling site and date in 2012 and 2013; Table S5: Table S5. Proportion *of Aproceros leucopoda* larvae, subdivided into early, middle and old instars, and pupae, subdivided into early and old pupae, corresponding to newly formed pupae of green colour and older pupae of black colour, respectively, recorded per sampling site and date in 2012 and 2013.

Table S1. Criteria used to define the phenology and number of generations of *Aproceros leucopoda* based on field observations of eggs, larvae, pupae, and adults, supported by published developmental data.

Life stage/event	Criterion used to define phenology
Overwintering eonymphs	The last presence of diapausing eonymphs is assumed to coincide with the last capture of ovipositing females in early spring
Start of oviposition (1st generation)	Detection of the first eggs
Start of oviposition (subsequent generations)	Reappearance of eggs after at least one sampling date without eggs
End of egg hatching (each generation)	Eggs observed on the sampling date following the last observation of first-instar larvae
Larval period (each generation)	Interval from the detection of the first hatched larva to that of the last mature larva
Pupal period (each generation)	From the first detection or an increase in the abundance of newly formed pupae (green individuals in the cocoon) and older pupae (black individuals in the cocoon) following a sampling date in which mature larvae were recorded
Adult period (each generation)	Adults observed in the interval from the occurrence of pupae in the previous sampling to the absence of pupae in the previous sampling or by the absence of adult captures in the subsequent sampling

Table S2. Mean monthly temperature and rainfall data from April to September (2012–2013) obtained from a weather station located near the study sites 1 and 2 (weather station Udine S.O., https://www.osmer.fvg.it/clima.php?ln=, accessed on 7 May 2025); dd: number of days with daily rainfall greater than or equal to 5 mm / 1 mm

Month	Mean temperature (°C)			Rainfall (mm)			Rainfall (dd)		
	2012	2013	1990-2024	2012	2013	1990-2024	2012	2013	1990-2024
April	15.0	13.8	12.9	239.3	67.5	117.0	12 / 15	5 / 11	6 / 10
May	17.6	15.8	17.5	87.7	299.4	137.0	6 / 11	17 / 20	7 / 11
June	22.3	21.0	21.6	114.3	63.9	121.0	3 / 8	5 / 8	6 / 9
July	24.4	25.6	23.5	72.2	47.4	114.0	4 / 8	2 / 3	5 / 9
August	24.8	24.3	23.2	94.0	94.6	119.0	2 / 5	8 / 10	6 / 8
September	19.7	18.7	18.6	201.3	302.4	170.0	6 / 9	6 / 9	6 / 9

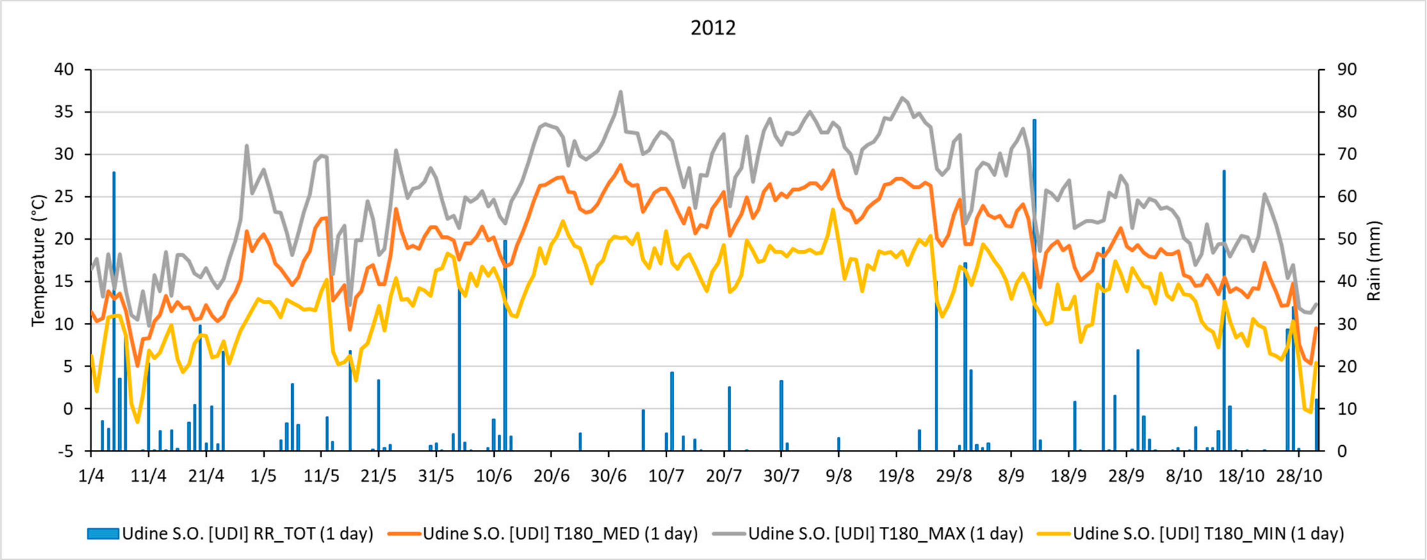

a)

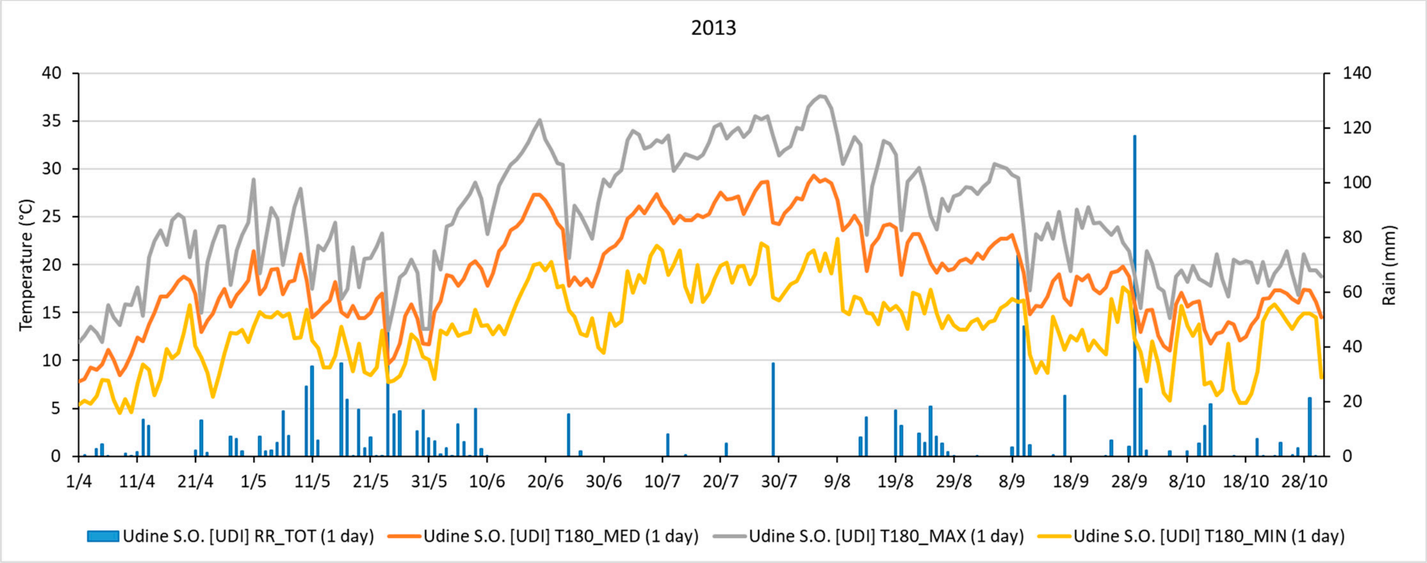

b)

Figure S1. Daily temperature and rainfall from April to October in **a)** 2012 and **b)** 2013, obtained from a weather station located near the study sites 1 and 2 (weather station Udine S.O., https://www.osmer.fvg.it/clima.php?ln=, accessed on 7 May 2025).

Table S3. Number of *Aproceros leucopoda* adults captured on two sticky traps per sampling site and date in 2012 and 2013.

Site	Location	Year	from date	to date	No. of Adults on Trap T1	No. of Adults on Trap T2	Sum of Adults on Traps T1 + T2	Avarage
1	Reana del Rojale (UD)	2012	12/04/2012	19/04/2012	62	4	66	33
1	Reana del Rojale (UD)	2012	19/04/2012	26/04/2012	10	2	12	6
1	Reana del Rojale (UD)	2012	26/04/2012	03/05/2012	5	1	6	3
1	Reana del Rojale (UD)	2012	03/05/2012	10/05/2012	0	1	1	0,5
1	Reana del Rojale (UD)	2012	10/05/2012	17/05/2012	0	0	0	0
1	Reana del Rojale (UD)	2012	17/05/2012	24/05/2012	8	0	8	4
1	Reana del Rojale (UD)	2012	24/05/2012	31/05/2012	3	2	5	2,5
1	Reana del Rojale (UD)	2012	31/05/2012	07/06/2012	1	0	1	0,5
1	Reana del Rojale (UD)	2012	07/06/2012	14/06/2012	1	0	1	0,5
1	Reana del Rojale (UD)	2012	14/06/2012	21/06/2012	33	6	39	19,5
1	Reana del Rojale (UD)	2012	21/06/2012	28/06/2012	23	20	43	21,5
1	Reana del Rojale (UD)	2012	28/06/2012	05/07/2012	5	2	7	3,5
1	Reana del Rojale (UD)	2012	05/07/2012	12/07/2012	128	52	180	90
1	Reana del Rojale (UD)	2012	12/07/2012	19/07/2012	104	47	151	75,5
1	Reana del Rojale (UD)	2012	19/07/2012	26/07/2012	2	0	2	1
1	Reana del Rojale (UD)	2012	26/07/2012	02/08/2012	211	12	223	111,5
1	Reana del Rojale (UD)	2012	02/08/2012	09/08/2012	80	38	118	59
1	Reana del Rojale (UD)	2012	09/08/2012	16/08/2012	0	0	0	0
1	Reana del Rojale (UD)	2012	16/08/2012	23/08/2012	0	0	0	0
1	Reana del Rojale (UD)	2012	23/08/2012	30/08/2012	0	0	0	0
1	Reana del Rojale (UD)	2012	30/08/2012	06/09/2012	0	0	0	0
1	Reana del Rojale (UD)	2012	06/09/2012	13/09/2012	0	0	0	0
1	Reana del Rojale (UD)	2012	13/09/2012	20/09/2012	0	0	0	0
1	Reana del Rojale (UD)	2012	20/09/2012	27/09/2012	0	0	0	0
1	Reana del Rojale (UD)	2012	27/09/2012	04/10/2012	0	0	0	0
1	Reana del Rojale (UD)	2012	04/10/2012	11/10/2012	0	0	0	0
1	Reana del Rojale (UD)	2012	11/10/2012	18/10/2012	0	0	0	0
1	Reana del Rojale (UD)	2012	18/10/2012	25/10/2012	0	0	0	0
2	Martignacco (UD)	2012	12/04/2012	19/04/2012	27	330	357	178,5
2	Martignacco (UD)	2012	19/04/2012	26/04/2012	2	26	28	14
2	Martignacco (UD)	2012	26/04/2012	03/05/2012	2	3	5	2,5
2	Martignacco (UD)	2012	03/05/2012	10/05/2012	0	1	1	0,5
2	Martignacco (UD)	2012	10/05/2012	17/05/2012	0	0	0	0
2	Martignacco (UD)	2012	17/05/2012	24/05/2012	1	24	25	12,5
2	Martignacco (UD)	2012	24/05/2012	31/05/2012	5	28	33	16,5
2	Martignacco (UD)	2012	31/05/2012	07/06/2012	0	2	2	1
2	Martignacco (UD)	2012	07/06/2012	14/06/2012	0	0	0	0
2	Martignacco (UD)	2012	14/06/2012	21/06/2012	147	474	621	310,5
2	Martignacco (UD)	2012	21/06/2012	28/06/2012	31	85	116	58
2	Martignacco (UD)	2012	28/06/2012	05/07/2012	10	7	17	8,5
2	Martignacco (UD)	2012	05/07/2012	12/07/2012	196	314	510	255
2	Martignacco (UD)	2012	12/07/2012	19/07/2012	28	24	52	26
2	Martignacco (UD)	2012	19/07/2012	26/07/2012	2	1	3	1,5
2	Martignacco (UD)	2012	26/07/2012	02/08/2012	3	16	19	9,5
2	Martignacco (UD)	2012	02/08/2012	09/08/2012	25	26	51	25,5
2	Martignacco (UD)	2012	09/08/2012	16/08/2012	0	0	0	0
2	Martignacco (UD)	2012	16/08/2012	23/08/2012	0	0	0	0
2	Martignacco (UD)	2012	23/08/2012	30/08/2012	0	0	0	0
2	Martignacco (UD)	2012	30/08/2012	06/09/2012	0	0	0	0
2	Martignacco (UD)	2012	06/09/2012	13/09/2012	0	0	0	0
2	Martignacco (UD)	2012	13/09/2012	20/09/2012	0	0	0	0
2	Martignacco (UD)	2012	20/09/2012	27/09/2012	0	0	0	0
2	Martignacco (UD)	2012	27/09/2012	04/10/2012	0	0	0	0
2	Martignacco (UD)	2012	04/10/2012	11/10/2012	0	0	0	0
2	Martignacco (UD)	2012	11/10/2012	18/10/2012	0	0	0	0
2	Martignacco (UD)	2012	18/10/2012	25/10/2012	0	0	0	0
1	Reana del Rojale (UD)	2013	12/04/2013	14/04/2013	0	0	0	0
1	Reana del Rojale (UD)	2013	14/04/2013	21/04/2013	41	3	44	22
1	Reana del Rojale (UD)	2013	21/04/2013	28/04/2013	0	6	6	3
1	Reana del Rojale (UD)	2013	28/04/2013	05/05/2013	0	0	0	0
1	Reana del Rojale (UD)	2013	05/05/2013	12/05/2013	0	0	0	0
1	Reana del Rojale (UD)	2013	12/05/2013	20/05/2013	0	0	0	0
1	Reana del Rojale (UD)	2013	20/05/2013	27/05/2013	3	0	3	1,5
1	Reana del Rojale (UD)	2013	27/05/2013	03/06/2013	2	0	2	1
1	Reana del Rojale (UD)	2013	03/06/2013	10/06/2013	0	0	0	0
1	Reana del Rojale (UD)	2013	10/06/2013	17/06/2013	0	0	0	0
1	Reana del Rojale (UD)	2013	17/06/2013	24/06/2013	24	10	34	17
1	Reana del Rojale (UD)	2013	24/06/2013	01/07/2013	23	13	36	18
1	Reana del Rojale (UD)	2013	01/07/2013	08/07/2013	6	5	11	5,5
1	Reana del Rojale (UD)	2013	08/07/2013	15/07/2013	12	6	18	9
1	Reana del Rojale (UD)	2013	15/07/2013	22/07/2013	22	2	24	12
1	Reana del Rojale (UD)	2013	22/07/2013	29/07/2013	6	0	6	3
1	Reana del Rojale (UD)	2013	29/07/2013	05/08/2013	0	0	0	0
1	Reana del Rojale (UD)	2013	05/08/2013	12/08/2013	1	0	1	1
1	Reana del Rojale (UD)	2013	12/08/2013	19/08/2013	0	0	0	0
1	Reana del Rojale (UD)	2013	19/08/2013	26/08/2013	0	0	0	0
1	Reana del Rojale (UD)	2013	26/08/2013	02/09/2013	0	0	0	0
1	Reana del Rojale (UD)	2013	02/09/2013	09/09/2013	0	0	0	0
1	Reana del Rojale (UD)	2013	09/09/2013	16/09/2013	0	0	0	0
1	Reana del Rojale (UD)	2013	16/09/2013	23/09/2013	0	0	0	0
1	Reana del Rojale (UD)	2013	23/09/2013	30/09/2013	0	0	0	0
1	Reana del Rojale (UD)	2013	30/09/2013	07/10/2013	0	0	0	0
1	Reana del Rojale (UD)	2013	07/10/2013	14/10/2013	0	0	0	0
1	Reana del Rojale (UD)	2013	14/10/2013	21/10/2013	0	0	0	0
1	Reana del Rojale (UD)	2013	21/10/2013	28/10/2013	0	0	0	0
2	Martignacco (UD)	2013	12/04/2013	14/04/2013	0	2	2	2
2	Martignacco (UD)	2013	14/04/2013	21/04/2013	62	197	259	129,5
2	Martignacco (UD)	2013	21/04/2013	28/04/2013	4	0	4	2
2	Martignacco (UD)	2013	28/04/2013	05/05/2013	0	0	0	0
2	Martignacco (UD)	2013	05/05/2013	12/05/2013	0	0	0	0
2	Martignacco (UD)	2013	12/05/2013	20/05/2013	1	1	2	1
2	Martignacco (UD)	2013	20/05/2013	27/05/2013	1	3	4	2
2	Martignacco (UD)	2013	27/05/2013	03/06/2013	1	0	1	0,5
2	Martignacco (UD)	2013	03/06/2013	10/06/2013	0	0	0	0
2	Martignacco (UD)	2013	10/06/2013	17/06/2013	1	0	1	0,5
2	Martignacco (UD)	2013	17/06/2013	24/06/2013	54	65	119	59,5
2	Martignacco (UD)	2013	24/06/2013	01/07/2013	23	5	28	14
2	Martignacco (UD)	2013	01/07/2013	08/07/2013	5	10	15	7,5
2	Martignacco (UD)	2013	08/07/2013	15/07/2013	53	14	67	33,5
2	Martignacco (UD)	2013	15/07/2013	22/07/2013	138	262	400	200
2	Martignacco (UD)	2013	22/07/2013	29/07/2013	2	20	22	11
2	Martignacco (UD)	2013	29/07/2013	05/08/2013	29	83	112	56
2	Martignacco (UD)	2013	05/08/2013	12/08/2013	8	8	16	8
2	Martignacco (UD)	2013	12/08/2013	19/08/2013	0	0	0	0
2	Martignacco (UD)	2013	19/08/2013	26/08/2013	0	0	0	0
2	Martignacco (UD)	2013	26/08/2013	02/09/2013	0	0	0	0
2	Martignacco (UD)	2013	02/09/2013	09/09/2013	0	0	0	0
2	Martignacco (UD)	2013	09/09/2013	16/09/2013	0	0	0	0
2	Martignacco (UD)	2013	16/09/2013	23/09/2013	0	0	0	0
2	Martignacco (UD)	2013	23/09/2013	30/09/2013	0	0	0	0
2	Martignacco (UD)	2013	30/09/2013	07/10/2013	0	0	0	0
2	Martignacco (UD)	2013	07/10/2013	14/10/2013	0	0	0	0
2	Martignacco (UD)	2013	14/10/2013	21/10/2013	0	0	0	0
2	Martignacco (UD)	2013	21/10/2013	28/10/2013	0	0	0	0

Table S4. Number of *Aproceros leucopoda* eggs, larvae, and pupae recorded, as total and average with standard deviation (SD) on 500 leaves per sampling site and date in 2012 and 2013.

				Eggs			Larvae			Pupae						
Site	Location	Year	Date	Sum of No. Eggs	Average of No. Eggs	SD of No. Eggs	Sum of No. Larvae	Average of No. Larvae	SD of No. Larvae	Sum of No. Pupae	Average of No. Pupae	SD of No. Pupae	Sum of No. of leaves with eggs	Sum of No. of leaves with larvae	Sum of No. of leaves with pupae	Sum of No. of leaves with eggs, larvae and pupae
1	Reana del Rojale (UD)	2012	12/04/2012	263	0,526	1,291766745	0	0	0	0	0	0	107	0	0	107
1	Reana del Rojale (UD)	2012	19/04/2012	386	0,772	1,642379622	0	0	0	0	0	0	142	0	0	142
1	Reana del Rojale (UD)	2012	26/04/2012	299	0,598	1,408674813	11	0,022	0,222744765	0	0	0	124	6	0	128
1	Reana del Rojale (UD)	2012	03/05/2012	0	0	0	177	0,354	0,713935156	0	0	0	0	121	0	121
1	Reana del Rojale (UD)	2012	10/05/2012	0	0	0	122	0,244	0,465722874	69	0,138	0,373141352	0	115	64	174
1	Reana del Rojale (UD)	2012	17/05/2012	0	0	0	2	0,004	0,063182149	83	0,166	0,41808323	0	2	75	77
1	Reana del Rojale (UD)	2012	24/05/2012	21	0,042	0,304007963	2	0,004	0,089442719	1	0,002	0,04472136	13	1	1	15
1	Reana del Rojale (UD)	2012	31/05/2012	40	0,08	0,337383543	110	0,22	0,565897942	0	0	0	32	80	0	100
1	Reana del Rojale (UD)	2012	07/06/2012	0	0	0	136	0,272	0,56449385	0	0	0	0	111	0	111
1	Reana del Rojale (UD)	2012	14/06/2012	0	0	0	112	0,224	0,467183605	83	0,166	0,488796321	0	101	67	167
1	Reana del Rojale (UD)	2012	21/06/2012	96	0,192	0,69288974	60	0,12	0,466943329	34	0,068	0,251998028	45	42	34	115
1	Reana del Rojale (UD)	2012	28/06/2012	17	0,034	0,270166578	233	0,466	0,744279453	4	0,008	0,109361418	10	170	4	177
1	Reana del Rojale (UD)	2012	05/07/2012	3	0,006	0,077304281	86	0,172	0,4321916	120	0,24	0,5431298	3	76	100	166
1	Reana del Rojale (UD)	2012	12/07/2012	736	1,472	3,186459328	109	0,218	0,838977811	40	0,08	0,285943223	132	48	38	175
1	Reana del Rojale (UD)	2012	19/07/2012	68	0,136	0,816601295	221	0,442	0,881015537	1	0,002	0,04472136	22	149	1	163
1	Reana del Rojale (UD)	2012	26/07/2012	0	0	0	32	0,064	0,316392491	55	0,11	0,409623709	0	25	45	65
1	Reana del Rojale (UD)	2012	02/08/2012	173	0,346	1,301411289	2	0,004	0,063182149	24	0,048	0,248635555	44	2	20	66
1	Reana del Rojale (UD)	2012	09/08/2012	91	0,182	0,985300784	210	0,42	1,254890234	0	0	0	25	97	0	110
1	Reana del Rojale (UD)	2012	16/08/2012	0	0	0	32	0,064	0,260844935	2	0,004	0,063182149	0	30	2	31
1	Reana del Rojale (UD)	2012	23/08/2012	0	0	0	0	0	0	1	0,002	0,04472136	0	0	1	1
1	Reana del Rojale (UD)	2012	30/08/2012	0	0	0	0	0	0	0	0	0	0	0	0	0
1	Reana del Rojale (UD)	2012	06/09/2012	0	0	0	0	0	0	0	0	0	0	0	0	0
1	Reana del Rojale (UD)	2012	13/09/2012	0	0	0	0	0	0	0	0	0	0	0	0	0
1	Reana del Rojale (UD)	2012	20/09/2012	0	0	0	0	0	0	0	0	0	0	0	0	0
1	Reana del Rojale (UD)	2012	27/09/2012	0	0	0	0	0	0	0	0	0	0	0	0	0
2	Martignacco (UD)	2013	14/04/2013	0	0	0	0	0	0	0	0	0	0	0	0	0
2	Martignacco (UD)	2013	21/04/2013	221	0,442	1,526859063	0	0	0	0	0	0	70	0	0	70
2	Martignacco (UD)	2013	28/04/2013	18	0,036	0,258529835	385	0,77	1,521094164	0	0	0	11	155	0	160
2	Martignacco (UD)	2013	05/05/2013	0	0	0	156	0,312	0,625650763	0	0	0	0	124	0	124
2	Martignacco (UD)	2013	12/05/2013	0	0	0	18	0,036	0,206856422	51	0,102	0,309495679	0	16	50	65
2	Martignacco (UD)	2013	20/05/2013	12	0,024	0,217988619	1	0,002	0,04472136	2	0,004	0,063182149	7	1	2	10
2	Martignacco (UD)	2013	27/05/2013	0	0	0	0	0	0	0	0	0	0	0	0	0
2	Martignacco (UD)	2013	03/06/2013	15	0,03	0,255402547	111	0,222	0,744193286	0	0	0	10	53	0	63
2	Martignacco (UD)	2013	10/06/2013	0	0	0	102	0,204	0,625432916	0	0	0	0	61	0	61
2	Martignacco (UD)	2013	17/06/2013	3	0,006	0,077304281	6	0,012	0,10899431	54	0,108	0,337828738	3	6	51	60
2	Martignacco (UD)	2013	24/06/2013	202	0,404	1,184732536	10	0,02	0,153776769	2	0,004	0,063182149	75	9	2	82
2	Martignacco (UD)	2013	01/07/2013	1	0,002	0,04472136	202	0,404	0,746926904	1	0,002	0,063182149	1	144	1	145
2	Martignacco (UD)	2013	08/07/2013	0	0	0	92	0,184	0,45005455	141	0,282	0,55415718	0	80	120	216
2	Martignacco (UD)	2013	15/07/2013	109	0,218	0,731810832	35	0,07	0,263132052	52	0,104	0,406835384	64	34	44	130
2	Martignacco (UD)	2013	22/07/2013	181	0,362	0,829658162	551	1,102	1,75435216	0	0	0	114	229	0	279
2	Martignacco (UD)	2013	29/07/2013	0	0	0	218	0,436	0,806973613	9	0,018	0,13308427	0	162	9	171
2	Martignacco (UD)	2013	05/08/2013	0	0	0	0	0	0	69	0,138	0,441980422	0	0	51	51
2	Martignacco (UD)	2013	12/08/2013	0	0	0	0	0	0	0	0	0	0	0	0	0
2	Martignacco (UD)	2013	19/08/2013	0	0	0	0	0	0	0	0	0	0	0	0	0
2	Martignacco (UD)	2013	26/08/2013	0	0	0	0	0	0	0	0	0	0	0	0	0
2	Martignacco (UD)	2013	02/09/2013	0	0	0	0	0	0	0	0	0	0	0	0	0
2	Martignacco (UD)	2013	09/09/2013	0	0	0	0	0	0	0	0	0	0	0	0	0
2	Martignacco (UD)	2013	16/09/2013	0	0	0	0	0	0	0	0	0	0	0	0	0
2	Martignacco (UD)	2013	23/09/2013	0	0	0	0	0	0	0	0	0	0	0	0	0
2	Martignacco (UD)	2013	30/09/2013	0	0	0	0	0	0	0	0	0	0	0	0	0

Table S5. Table S5. Proportion *of Aproceros leucopoda* larvae, subdivided into early, middle and old instars, and pupae, subdivided into early and old pupae, corresponding to newly formed pupae of green colour and older pupae of black colour, respectively, recorded per sampling site and date in 2012 and 2013.

				Larvae							Pupae				
Site	Location	Year	Date	No. of Larvae	Early instar larvae (L1 and L2)	Middle instar larvae (L3-L4)	Old instar larvae (L5 and L6)	% Early instar larvae (L1 and L2)	% Middle instar larvae (L3-L4)	% Old instar larvae (L5 and L6)	No. of Pupae	Early pupa (of green colour)	Old pupae (of black colour)	% Early pupa (of green colour)	% Old pupae (of black colour)
1	Reana del Rojale (UD	2012	12/04/2012	0	0	0	0	0,00	0,00	0,00	0	0	0	0,00	0,00
1	Reana del Rojale (UD	2012	19/04/2012	0	0	0	0	0,00	0,00	0,00	0	0	0	0,00	0,00
1	Reana del Rojale (UD	2012	26/04/2012	11	11	0	0	100,00	0,00	0,00	0	0	0	0,00	0,00
1	Reana del Rojale (UD	2012	03/05/2012	177	18	159	0	10,17	89,83	0,00	0	0	0	0,00	0,00
1	Reana del Rojale (UD	2012	10/05/2012	122	4	15	103	3,28	12,30	84,43	69	69	0	100,00	0,00
1	Reana del Rojale (UD	2012	17/05/2012	2	0	0	2	0,00	0,00	100,00	83	77	6	92,77	7,23
1	Reana del Rojale (UD	2012	24/05/2012	2	2	0	0	100,00	0,00	0,00	2	1	1	50,00	50,00
1	Reana del Rojale (UD	2012	31/05/2012	110	101	9	0	91,82	8,18	0,00	0	0	0	0,00	0,00
1	Reana del Rojale (UD	2012	07/06/2012	136	70	50	16	51,47	36,76	11,76	0	0	0	0,00	0,00
1	Reana del Rojale (UD	2012	14/06/2012	112	29	48	35	25,89	42,86	31,25	84	83	1	98,81	1,19
1	Reana del Rojale (UD	2012	21/06/2012	60	49	8	3	81,67	13,33	5,00	34	14	20	41,18	58,82
1	Reana del Rojale (UD	2012	28/06/2012	233	130	73	30	55,79	31,33	12,88	4	4	0	100,00	0,00
1	Reana del Rojale (UD	2012	05/07/2012	86	7	20	59	8,14	23,26	68,60	120	97	23	80,83	19,17
1	Reana del Rojale (UD	2012	12/07/2012	109	107	0	2	98,17	0,00	1,83	40	25	15	62,50	37,50
1	Reana del Rojale (UD	2012	19/07/2012	221	153	67	1	69,23	30,32	0,45	1	0	1	0,00	100,00
1	Reana del Rojale (UD	2012	26/07/2012	32	7	5	20	21,88	15,63	62,50	57	55	2	96,49	3,51
1	Reana del Rojale (UD	2012	02/08/2012	2	1	1	0	50,00	50,00	0,00	24	17	7	70,83	29,17
1	Reana del Rojale (UD	2012	09/08/2012	210	158	52	0	75,24	24,76	0,00	0	0	0	0,00	0,00
1	Reana del Rojale (UD	2012	16/08/2012	32	13	9	10	40,63	28,13	31,25	2	2	0	100,00	0,00
1	Reana del Rojale (UD	2012	23/08/2012	0	0	0	0	0,00	0,00	0,00	1	0	1	0,00	100,00
1	Reana del Rojale (UD	2012	30/08/2012	0	0	0	0	0,00	0,00	0,00	0	0	0	0,00	0,00
1	Reana del Rojale (UD	2012	06/09/2012	0	0	0	0	0,00	0,00	0,00	0	0	0	0,00	0,00
1	Reana del Rojale (UD	2012	13/09/2012	0	0	0	0	0,00	0,00	0,00	0	0	0	0,00	0,00
1	Reana del Rojale (UD	2012	20/09/2012	0	0	0	0	0,00	0,00	0,00	0	0	0	0,00	0,00
1	Reana del Rojale (UD	2012	27/09/2012	0	0	0	0	0,00	0,00	0,00	0	0	0	0,00	0,00
2	Martignacco (UD)	2013	14/04/2013	0	0	0	0	0,00	0,00	0,00	0	0	0	0,00	0,00
2	Martignacco (UD)	2013	21/04/2013	0	0	0	0	0,00	0,00	0,00	0	0	0	0,00	0,00
2	Martignacco (UD)	2013	28/04/2013	385	280	105	0	72,73	27,27	0,00	0	0	0	0,00	0,00
2	Martignacco (UD)	2013	05/05/2013	156	0	98	58	0,00	62,82	37,18	0	0	0	0,00	0,00
2	Martignacco (UD)	2013	12/05/2013	18	0	0	18	0,00	0,00	100,00	51	51	0	100,00	0,00
2	Martignacco (UD)	2013	20/05/2013	1	0	0	1	0,00	0,00	100,00	2	0	2	0,00	100,00
2	Martignacco (UD)	2013	27/05/2013	0	0	0	0	0,00	0,00	0,00	0	0	0	0,00	0,00
2	Martignacco (UD)	2013	03/06/2013	111	97	14	0	87,39	12,61	0,00	0	0	0	0,00	0,00
2	Martignacco (UD)	2013	10/06/2013	102	0	30	72	0,00	29,41	70,59	0	0	0	0,00	0,00
2	Martignacco (UD)	2013	17/06/2013	6	0	0	6	0,00	0,00	100,00	55	32	23	58,18	41,82
2	Martignacco (UD)	2013	24/06/2013	10	2	0	8	20,00	0,00	80,00	2	0	2	0,00	100,00
2	Martignacco (UD)	2013	01/07/2013	202	20	60	122	9,90	29,70	60,40	2	0	2	0,00	100,00
2	Martignacco (UD)	2013	08/07/2013	92	0	19	73	0,00	20,65	79,35	141	140	1	99,29	0,71
2	Martignacco (UD)	2013	15/07/2013	35	30	5	0	85,71	14,29	0,00	52	23	29	44,23	55,77
2	Martignacco (UD)	2013	22/07/2013	551	314	237	0	56,99	43,01	0,00	0	0	0	0,00	0,00
2	Martignacco (UD)	2013	29/07/2013	218	0	165	53	0,00	75,69	24,31	9	9	0	100,00	0,00
2	Martignacco (UD)	2013	05/08/2013	0	0	0	0	0,00	0,00	0,00	69	53	16	76,81	23,19
2	Martignacco (UD)	2013	12/08/2013	0	0	0	0	0,00	0,00	0,00	0	0	0	0,00	0,00
2	Martignacco (UD)	2013	19/08/2013	0	0	0	0	0,00	0,00	0,00	0	0	0	0,00	0,00
2	Martignacco (UD)	2013	26/08/2013	0	0	0	0	0,00	0,00	0,00	0	0	0	0,00	0,00
2	Martignacco (UD)	2013	02/09/2013	0	0	0	0	0,00	0,00	0,00	0	0	0	0,00	0,00
2	Martignacco (UD)	2013	09/09/2013	0	0	0	0	0,00	0,00	0,00	0	0	0	0,00	0,00
2	Martignacco (UD)	2013	16/09/2013	0	0	0	0	0,00	0,00	0,00	0	0	0	0,00	0,00
2	Martignacco (UD)	2013	23/09/2013	0	0	0	0	0,00	0,00	0,00	0	0	0	0,00	0,00
2	Martignacco (UD)	2013	30/09/2013	0	0	0	0	0,00	0,00	0,00	0	0	0	0,00	0,00
